# Intra-Herb Interactions: Primary Metabolites in *Coptidis Rhizoma* Extract Improved the Pharmacokinetics of Oral Berberine Hydrochloride in Mice

**DOI:** 10.3389/fphar.2021.675368

**Published:** 2021-06-07

**Authors:** Jing Zhao, Ting Zhou, Jing-Ze Lu, Dan Ye, Sheng Mu, Xin-Hui Tian, Wei-Dong Zhang, Bing-Liang Ma

**Affiliations:** ^1^ Department of Pharmacology, School of Pharmacy, Shanghai University of Traditional Chinese Medicine, Shanghai, China; ^2^ Institute of Interdisciplinary Integrative Medicine Research, Shanghai University of Traditional Chinese Medicine, Shanghai, China; ^3^ School of Pharmacy, Second Military Medical University, Shanghai, China

**Keywords:** natural deep eutectic solvent, intestinal absorption, berberine hydrochloride, *Coptidis Rhizoma*, malic acid, pharmacokinetics

## Abstract

Primary plant metabolites can be used for artificial preparation of natural deep eutectic solvents (NADESs), which have strong dissolving capacity, good biocompatibility, and biodegradability. In this study, for the first time, we verified that NADESs were present in *Coptidis Rhizoma* extract and systematically investigated its effects and mechanisms on the pharmacokinetics of oral berberine hydrochloride (BBR), a co-existing bioactive constituent. First, three LC-MS/MS based methods were established and fully validated to determine the levels of 11 primary metabolites in *Coptidis Rhizoma* extract. According to the weight ratio of four major primary metabolites in the *Coptidis Rhizoma* extract, a stable “endogenous” NADES was prepared using the heating method by the addition of 350 *μ*l of water to 1,307.8 mg of the mixture of malic acid (490.5 mg), glucose (280.6 mg), sucrose (517.7 mg), and choline chloride (19.0 mg). The prepared NADES showed significant acute toxicity in mice and cytotoxicity in MDCK-MDR1 cells. However, after being diluted 10 times or 100 times, the NADES had no significant acute toxicity or cytotoxicity, respectively. The dilutions of the NADES significantly increased the water solubility of BBR, reduced its efflux in gut sacs and MDCK-MDR1 cell monolayer, and improved its metabolic stability in intestinal S9. In addition, the NADES dilutions reversibly opened the tight junctions between the enterocytes in the gut sacs. Moreover, the NADES dilutions significantly improved the exposure levels of BBR in the portal vein and livers of mice that were administered oral BBR. Malic acid was identified as a major component in the NADES in terms of solubility, acute toxicity, cytotoxicity, and pharmacokinetic-improving effects on oral BBR. In conclusion, the primary metabolites of *Coptidis Rhizoma* extract could form “endogenous” NADES, and its dilutions improve the pharmacokinetics of oral BBR. This study demonstrates the synergistic interaction of the constituents of *Coptidis Rhizoma* extract and the potential use of the NADES dilutions in oral BBR delivery.

## Introduction

According to the Biopharmaceutics Classification System (BCS), water solubility is one of the fundamental parameters associated with the intestinal absorption of oral drugs (Amidon et al., 1995). Unfortunately, approximately 40% of marketed compounds and most drug candidates have poor solubility in water (Williams et al., 2013). Different strategies have been developed to improve drug solubility, including the use of deep eutectic solvents (DESs) (Zhang et al., 2012). DESs can be prepared by mixing hydrogen bond acceptors (HBAs) and hydrogen bond donors (HBDs) together at specific molar ratios (Abbott et al., 2004). HBAs are typically quaternary ammonium salts, such as choline chloride, whereas HBDs include urea, carboxylic acids (e.g., oxalic, citric, succinic, or amino acids), or polyols (e.g., glycerol or carbohydrates) (Zhang et al., 2012). In particular, DESs that are prepared using naturally occurring HBAs and HBDs, which are primarily plant metabolites, are known as “natural deep eutectic solvents” (NADESs) (Choi et al., 2011). NADESs are increasingly gaining research attention because of their biocompatibility, biodegradability, and especially, their strong solubilizing ability (Liu et al., 2018a). Compared with water, NADESs can increase the solubility of some lipophilic compounds, including those isolated from herbal extracts, by 18–460,000 folds (Dai et al., 2013).

Berberine hydrochloride (BBR) has a wide range of pharmacological effects, and it is the main active constituent of *Coptidis Rhizoma* “(*Coptis chinensis* Franch.)”, a commonly used traditional Chinese medicine (Kumar et al., 2015; Neag et al., 2018). However, the oral bioavailability of BBR is extremely low (0.36%) (Liu et al., 2010). Due to causes including poor water solubility, about 56% of oral BBR is directly removed with feces (Liu et al., 2010). In addition, intestinal efflux transporters, especially p-glycoprotein (P-gp) and intestinal drug metabolizing enzymes, cause elimination of about 43.5% of oral BBR (Liu et al., 2010). In contrast, it was found that the oral bioavailability of BBR in *Coptidis Rhizoma* extract was much higher than pure BBR (Ma et al., 2016b; Li et al., 2018), suggesting that some coexisting constituents in *Coptidis Rhizoma* extract improves the pharmacokinetic properties of oral BBR.

Using a mixture of sugars, amino acids, organic acids, and other nitrogen-containing compounds, several NADESs have been artificially prepared to increase the solubility and intestinal absorption of BBR (Sut et al., 2017). Interestingly, it was assumed that NADESs can be produced naturally by plants and are involved in the biosynthesis, storage, and transport of coexisting small molecules (Choi et al., 2011; Dai et al., 2013). However, given that NADESs are usually synthesized using no more than three components at integer stoichiometric ratios (Dai et al., 2013), it was not clear whether they are present in herbal extracts, where there are various components at varying ratios. In addition, it has been suggested that the high viscosity of NADESs may restrict domain motion related to the catalytic function of some enzymes and diffusion of substrates (Knudsen et al., 2020). This suggests that NADESs may substantially reduce the activity of metabolizing enzymes and transporters that are deeply involved in the metabolism and disposition of oral drugs. Therefore, in addition to solubility, the influence of “endogenous” NADESs on the intestinal permeability and intestinal metabolic stability of coexisting bioactive small molecules was worth exploring.

Therefore, in this study, we aimed to verify if NADESs could be produced from several major small primary metabolites in *Coptidis Rhizoma* extract. Subsequently, the influences and the related mechanisms of a prepared NADES on the pharmacokinetics of oral BBR in mice were investigated.

## Materials and Methods

### Materials

Herbal pieces of *Coptidis Rhizoma* “(*Coptis chinensis* Franch.)” (No.180305) were purchased from Shanghai Kang Qiao Herbal Pieces Co., Ltd. (Shanghai, China). The herbal pieces were identified as the root of *Coptis chinensis* Franch. according to *the Pharmacopeia of the People's Republic of China* (2015 edition). The purity of all the reference compounds used in this study was greater than 98%. BBR, coptisine, epiberberine, palmatine, demethyleneberberine, carbamazepine, verapamil hydrochloride, tetraethylamine, caffeic acid, and mycophenolic acid were purchased from Shanghai Yuanye Biological Co., Ltd. (Shanghai, China). Dimethyl sulfoxide, acetonitrile, and fluorescein isothiocyanate-dextran (FD4) were purchased from Merck and Co., Inc. (NJ, United States). Malic acid, sucrose, glucose, choline chloride, urea, fructose, proline, alanine, and glycine were obtained from China National Pharmaceutical Group Co., Ltd. (Shanghai, China). The BCA protein test kit was obtained from Shanghai Biyuntian Biotechnology Co., Ltd. (Shanghai, China). Pooled CD-1 mouse intestinal S9 fraction was obtained from SEKISUI Medical Co. Ltd. (Tokyo, Japan). Formic acid, ammonium formate, fetal bovine serum, and Dulbecco’s modified Eagle’s medium (DMEM) were purchased from Thermo Fisher Scientific (MA, United States). Cell Counting Kit-8 (CCK-8) was purchased from Meilunbio^®^ (Daliang, China). Trypsinase and penicillin-streptomycin solution were obtained from Biosharp (Hefei, China). The pure water used in the current study was prepared using a Millipore Milli-Q system (MA, United States).

### Cell Culture

Madin-Darby canine kidney cells stably expressing the transporter P-gp (MDCK-MDR1) were provided and authenticated by Sandia Pharmaceutical Technology (Shanghai) Co., Ltd. (Shanghai, China). The cells were cultured at 37°C in DMEM supplemented with 10% FBS, penicillin-streptomycin solution, and HEPES (15 mM) in a humidified atmosphere of 5% CO_2_. Hank’s balanced salt solution (HBSS) (consisting of 135 mM NaCl, 1.2 mM MgCl_2_, 0.81 mM MgSO_4_, 27.8 mM glucose, 2.5 mM CaCl_2_, and 25 mM HEPES, pH 7.2] was used to replace the culture medium in the incubation experiments. Where DMSO was used, its final concentration was restricted to less than 1‰.

### Animals

Grade II ICR mice (male and female, 22 ± 2 g body weight) were purchased from Shanghai Slac Laboratory Animal Co., Ltd. (Shanghai, China). The mice were housed in an air-conditioned room at 22–24°C with a dark/light cycle of 12 h. Before the experiment, the mice were fasted for approximately 12 h but were allowed to drink freely. All animal experimental protocols were approved by (PZSHUTCM19011105) and all the experiments were performed in accordance with the guidelines of the Institutional Animal Care and Use Committee of Shanghai University of Traditional Chinese Medicine.

### Preparation and Qualitative Analysis of *Coptidis Rhizoma* Extract

The herbal pieces of *Coptidis Rhizoma* were extracted twice with 10 times volume of boiling water (1.5 h for the first and 1 h for the second extraction). The obtained aqueous extract was then filtered through eight layers of gauze and transferred for vacuum drying at 60°C. The alkaloids in the dry *Coptidis Rhizoma* extract were quantified for quality control using a liquid chromatography-mass spectrometry (LC-MS) method (*Quantification of several Coptidis Rhizomaalkaloids*).

### Quantification of Several *Coptidis Rhizoma* Alkaloids

A validated method based on a liquid chromatography-mass spectrometry (LC-MS) system was used for the quantitative analysis of *Coptidis Rhizoma* alkaloids present in relatively high concentrations (0.156–10 *μ*g/ml). The LC-MS system was composed of a Shimadzu HPLC (LC-20AD) spectrometer (Kyoto, Japan) and a Thermo Scientific LCQ fleet mass spectrometer (MA, United States) and equipped with an electrospray ionization (ESI) source. An Eclipse XDB-C18 (4.6 × 150 mm, 5 *µ*m) maintained at room temperature was used for chromatographic separation of the analytes. Water containing formic acid (0.0625%) and ammonium formate (4 mM) was used as mobile phase A, and methanol was used as mobile phase B. The following elution gradient was used at the flow rate of 0.3 ml/min: 0–7 min, 20–30% B; 7.01–10 min, 20%–20% B. Data acquisition was performed in the selected ion monitoring mode after the protonated (M + H)^+^ ions of berberine and epiberberine (m/z 336.2, with different retention time), coptisine (m/z 320.2), palmatine (m/z 352.1), and carbamazepine (internal standard, m/z 237.0) were generated. The method was validated in terms of selectivity, sensitivity, linearity, intra-day and inter-day variation, and recovery (data not shown).

A validated method based on a liquid chromatography tandem mass spectrometry (LC-MS/MS) system was used for the quantitative analysis of BBR with relatively low concentrations (1.95–1,000 ng/ml) in biological samples. Further details about the method can be found elsewhere (Ma et al., 2012; Ma et al., 2017).

### Quantification of the Primary Metabolites in *Coptidis Rhizoma* Extract

Three LC-MS/MS methods were established and fully validated for the quantitative analysis of 15 primary metabolites in the *Coptidis Rhizoma* extract. The LC-MS/MS system consisted of a Waters ACQUITY ultra performance liquid chromatography system (MA, United States) and an API 5,500 mass spectrometer (Boston, MA, United States), and it was equipped with an ESI source.

For organic acids, an ACQUITY BEH C18 column (2.1 × 100 mm, 1.7 *μ*m) was used. Water with 0.1% formic acid was used as mobile phase A, while methanol was used as mobile phase B. Gradient elution was set as follows: 0–1 min: 5% B; 2–10 min: 5–40% B; 10–12 min: 40–90% B; 12–12.1 min: 90–5% B; 12.1–15 min: 5% B. The flow rate was 0.3 ml/min and the column temperature was maintained at 40°C. A 10-*μ*L aliquot was injected into the LC-MS/MS system for analysis. Negative ion mode was used for the ESI source, and data acquisition was performed in the multiple reaction monitoring (MRM) mode: *m/z* 132.9→114.9 for malic acid, *m/z* 179.2→135.1 for caffeic acid, and *m/z* 319.0→191.0 for mycophenolic acid (internal standard).

For sugars and amino acids, an ACQUITY BEH amide column (2.1 × 100 mm, 1.7 *μ*m) was used. Water containing 10 mM ammonium acetate was used as mobile phase A, while acetonitrile containing 10 mM ammonium acetate was used as mobile phase B. Gradient elution was set as follows: 0–6 min: 95%→60% B; 6–7 min: 60% B; 7–8 min: 60%→95% B; 8–10 min: 95% B. The flow rate was 0.3 ml/min, and the column temperature was maintained at 45°C. A 10-*μ*L aliquot was injected for analysis. Both positive and negative ion modes were used for the ESI source and data acquisition was performed in the MRM mode: *m/z* 150.9→88.7 for xylitol, *m/z* 179.0→88.9 for glucose and fructose, *m/z* 341.1→160.9 for sucrose, *m/z* 115.8→70.1 for proline, *m/z* 90.3→44.1 for alanine, *m/z* 76.2→30.0 for glycine, *m/z* 319.0→191.0 for mycophenolic acid (negative internal standard), and *m/z* 130.3→100.3 for tetraethylamine (positive internal standard).

For urea and choline chloride, an ACQUITY BEH amide column (2.1 × 100 mm, 1.7 *μ*m) was used. Water containing 0.1% formic acid was used as mobile phase A, while acetonitrile was used as mobile phase B. Gradient elution was set as 0–1 min: 95% B; 1–5 min: 95%→60% B; 5–6 min: 60%→95% B; 6–6.1 min: 60%→95% B; 6.1–8 min: 95% B. The flow rate was 0.3 ml/min, and the column temperature was maintained at 45°C. A 5-*μ*L aliquot was injected for analysis. Positive ion mode was used for the ESI source and data acquisition was performed in the MRM mode: *m/z* 61.0→44.0 for urea, *m/z* 104.2→60.1 for choline chloride, and *m/z* 130.3→100.3 for tetraethylamine (internal standard).

A methanol-water (50:50, v/v) solution of the *Coptidis Rhizoma* extract (1 mg/ml) was prepared and then treated with ultrasound for 60 min. After centrifugation at 20,000 g for 10 min, the supernatant was collected. After certain dilution, the supernatant was injected into the LC-MS/MS system for analysis.

### Preparation of Natural Deep Eutectic Solvents

NADESs were prepared by using the heating method (Dai et al., 2013). In brief, four major primary metabolites in *Coptidis Rhizoma* extract were mixed together according to different combinations. That is, two, three or four compounds of 490.5 mg malic acid, 280.6 mg glucose, 517.7 mg sucrose and 19.0 mg choline chloride were mixed together according to their contents in the extract (refer to *Quantification of the primary metabolites in Coptidis Rhizomaextract*: malic acid 28.14 mg/g, glucose 16.10 mg/g, sucrose 29.70 mg/g, choline chloride 1.09 mg/g, respectively). The mixtures were then, respectively, transferred to a glass bottle with lid. After 350 *μ*L water was, respectively, added, the mixtures were stirred and heated in a water bath at 50°C for 60 min to determine whether a transparent liquid, i.e., a NADES, was formed. The stability of the prepared NADESs was evaluated after placing them for 48 h at 20°C by observing whether the solutions were still transparent. In order to investigate the effect of water addition on the formation and stability of the prepared NADESs, 200, 250, 300, 350 or 400 *μ*L pure water was added into the mixture of the four metabolites (1,307.8 mg in total) to prepare four-component NADESs and evaluate their stability.

### Acute Toxicity in Mice

Sixty ICR mice were randomly divided into three groups according to gender and body weight, with 20 mice in each group. After fasting for 12 h, the mice were orally administered with 0.2 ml/10 g body weight the NADES (the four-component NADES prepared with 350 *μ*L water, the same below) or its 30 and 10% water dilutions, respectively. The symptoms of the mice were then observed, and the death time and the number of dead mice within 3 days were recorded.

The above method was also used to evaluate the acute toxicity of malic acid at different dosages, i.e., 9,800, 2,940, or 980 mg/kg, which were, respectively, equal to the dosages of malic acid in the NADES and its 30%, and10% water dilutions.

### Cytotoxicity in Madin-Darby Canine Kidney-Multidrug Resistance Gene-1 Cells

The cells were seeded into 96 well plate at the density of 3 × 10^4^ cells per well. After 24 h incubation in the incubator, the culture medium was carefully sucked out, and then 200 *μ*L DMEM medium containing 0.1, 0.3, 1 or 3% NADES was added. The control well was added with the same volume of DMEM medium. After incubation for 4 h or 24 h, 10% CCK-8 solution was added. After incubation for another 2 h, the absorbance at 450 nm was measured by using a microplate reader. The cytotoxicity of the NADES dilutions was evaluated by comparing the absorbance of the treatment groups to that of the control group.

The above method was also used to evaluate the cytotoxicity of different concentrations of malic acid, i.e., 4.90, 1.47 or 0.49 mg/ml, which were, respectively, equal to the concentrations of malic acid in the 1, 0.3 or 0.1% dilutions of the NADES.

### Influences on the Solubility of Berberine Hydrochloride

BBR was added to the prepared NADES or its 3, 10, or 30% water dilutions to produce a final concentration of 20 mg/ml. BBR was dissolved in pure water as a control. The solutions were ultrasonically treated for 1 h, and after full vortexing, the solutions were placed at room temperature for 1 h. Next, the solutions were centrifuged at 20,000 g for 10 min at 20°C. The obtained supernatant was filtered through a 0.22 *μ*m membrane. The filtered solution was diluted with a certain volume of methanol, followed by injection into the LC-MS system for the quantitative analysis of BBR (*Quantification of several Coptidis Rhizomaalkaloids*).

The above method was also used to evaluate the influences of solutions of malic acid, glucose, sucrose, and choline chloride on the solubility of BBR. The concentrations of the primary metabolites were equal to their concentrations in the NADES, i.e., 490.5 mg/ml, 280.6 mg/ml, 517.7 mg/ml, and 19.0 mg/ml, respectively. In addition, the influences of different concentrations (20, 40, 80, 160, 320, and 640 mg/ml) of malic acid on the solubility of BBR were also evaluated.

### Transportation in the Gut Sacs

The influence of NADES dilutions on the transportation of BBR was determined as previously described (Ma et al., 2012; Ma et al., 2017). For transportation from the mucosal side to the serosal side, the gut sac was filled on the inner mucosal side with 1 ml of Krebs-Ringer buffer containing 200 *μ*g/ml BBR in the absence or presence of 1, 3, or 10% NADES dilutions. For transportation from the serosal side to the mucosal side, the gut sac was everted and filled on the inner serosal side with 1 ml Krebs-Ringer buffer containing 200 *μ*g/ml BBR in the absence or presence of 1, 3, or 10% NADES dilutions. After incubation, aliquots of buffer (100 *μ*L) were, respectively, taken from the serosal side or mucosal side every 15–60 min and replaced with an equal volume of blank Krebs-Ringer buffer. The LC-MS system (*Quantification of several Coptidis Rhizomaalkaloids*) was used for the quantitative analysis of BBR.

Fluorescein isothiocyanate-labeled dextran (FD4) with a molecular weight of 4,400 g/moL, is usually used to test the integrity of intestinal paracellular tight junctions (Ma et al., 2012). The influence of various NADES dilutions (1, 3, and 10%) on the *in vitro* transportation of FD4 (3 mg/ml) across the mouse gut sac was determined. The concentrations of FD4 were determined using a fluorescence microplate reader (BioTek Instruments, Winooski, VT, United States), with excitation and emission at 485 and 528 nm, respectively, (Ma et al., 2012). In addition, the effects of NADES dilutions pretreatment on the transportation of FD4 across the mouse gut sacs were also determined. Several mice were orally administered with 10% NADES aqueous solution. The intestines were collected after 4 h, completely washed with blank Krebs-Ringer buffer, and subsequently used for the transportation study.

The above method was also used to evaluate the influences of different concentrations of malic acid on the transportation of BBR and FD4 in gut sacs. The concentrations of malic acid were 4.9, 14.7 and 49 mg/ml, which were respectively equal to the concentrations of malic acid in the 1, 3 or 10% water dilutions of the NADES.

### Transportation Across Madin-Darby Canine Kidney-Multidrug Resistance Gene-1 Cell Monolayer

MDCK-MDR1 cells were seeded on a Merck Transwell polycarbonate membrane (NJ, United States) at a density of 2 × 10^5^ cells/mL. The cells were cultured until the tight junctions were formed (transepithelial electrical resistance value >500 Ω cm2). Before the experiment, the media on both sides of the chamber were replaced with a warmed HBSS solution. The cells were then incubated for 20 min at 37°C. For the transportation test from the apical (AP) to the basolateral (BL) sides, 0.2 ml of HBSS solution containing BBR (10 *μ*g/ml) in the absence or presence of 0.3% or 1% of NADES was added to the AP side, and 0.7 ml blank HBSS solution was added to the BL side. For the transportation test from the BL side to the AP side, 0.2 ml blank HBSS solution was added to the AP side and 0.7 ml of HBSS solution containing BBR in the absence or presence of 0.3% or 1% of NADES was added to the BL side. After incubation for 2 or 4 h, the solutions on both sides were aspirated, and the concentration of BBR was determined using the LC-MS/MS method (*Quantification of several Coptidis Rhizomaalkaloids*).

### Metabolism in Intestinal Supernatant 9

BBR (10 *μ*g/ml) in the absence or presence of 0.3% or 1% of NADES was mixed with intestinal S9 (2 mg/ml) in 100 *μ*L Tris-HCl (50 mM, pH 7.4) buffer solution and then pre-incubated at 37°C for 5 min. Next, reduced nicotinamide adenine dinucleotide phosphate (NADPH, 1 mg/ml) was added to initiate the reaction. After 0.5 or 1 h, the incubation was terminated with the same amount of chilled methanol, which contained carbamazepine as the internal standard. After centrifugation at 20,000 g for 6 min, the concentration of demethylberberine, a representative metabolite of BBR, in the obtained supernatant was determined using the LC-MS/MS method (*Quantification of several Coptidis Rhizomaalkaloids*). The metabolic stability of BBR was characterized by using the concentration of demethylberberine.

### Pharmacokinetics in Mice

Mice were randomly divided into five groups, and they were orally administered with BBR that were dissolved in water, water dilutions of the NADES (1 and 10%), or solutions of malic acid (98 or 980 mg/kg). The dosages of malic acid were, respectively, equal to the dosages of malic acid in the 1 and 10% water dilutions of the NADES. The dosage of BBR in each group was 200 mg/kg, which was consistent with the dosage (0.1–0.3 g/kg) reported in previous studies on mice (Liu et al., 2016). Six mice in each group were anesthetized using diethyl ether at 0.25, 0.5, 1, 2, 3, 4, 6, 8, or 12 h after administration. Blood samples were then collected from the portal vein into heparin-containing tubes. The plasma samples were obtained by centrifugation of the blood samples at 1,000 g for 10 min at 4°C. The livers of the mice were collected and homogenized in 10 × volume of water. At the end of the experiment, the mice were euthanized by cervical dislocation. The plasma and liver homogenate samples were stored at -80°C, and the concentration of BBR in each sample was determined using the LC-MS/MS method (*Quantification of several Coptidis Rhizomaalkaloids*).

### Data Analysis

The permeability (*P*_*app*_) of BBR in the MDCK-MDR1 experiment was calculated using the equation: *P*_*app*_ = ΔQ/(Δt × A × C_0_), where ΔQ is the drug amount transported within Δt time, A is the surface area (0.33 cm^2^ in this experiment), and C_0_ is the initial concentration of BBR on the donor side. The unit of *P*_*app*_ is cm/sec.

The efflux rate (ER) of BBR in the MDCK-MDR1 experiment was calculated using the following equation: ER = *P*_*app*_ _*(BL-AP)*_/*P*_*app (AP-BL)*_.

A non-compartmental analysis using WinNonlin® software (Pharsight, CA, United States) was performed to obtain the pharmacokinetic parameters of berberine in mice. It should be noted that the pharmacokinetic parameters were calculated based on the average drug concentration at each time point because the mice were not continuously sampled.

The results are expressed as Mean ± SD, and statistical significance was determined with one-way or two-way analysis of variance (ANOVA) for multiple comparisons. A *p* value < 0.05 indicated statistical significance and a *p* value < 0.01 indicated high statistical significance.

## Results

### Validation of the Liquid Chromatography Tandem Mass Spectrometry Methods for Determining the Primary Metabolites in *Coptidis Rhizoma* Extract

**FIGURE 1:**
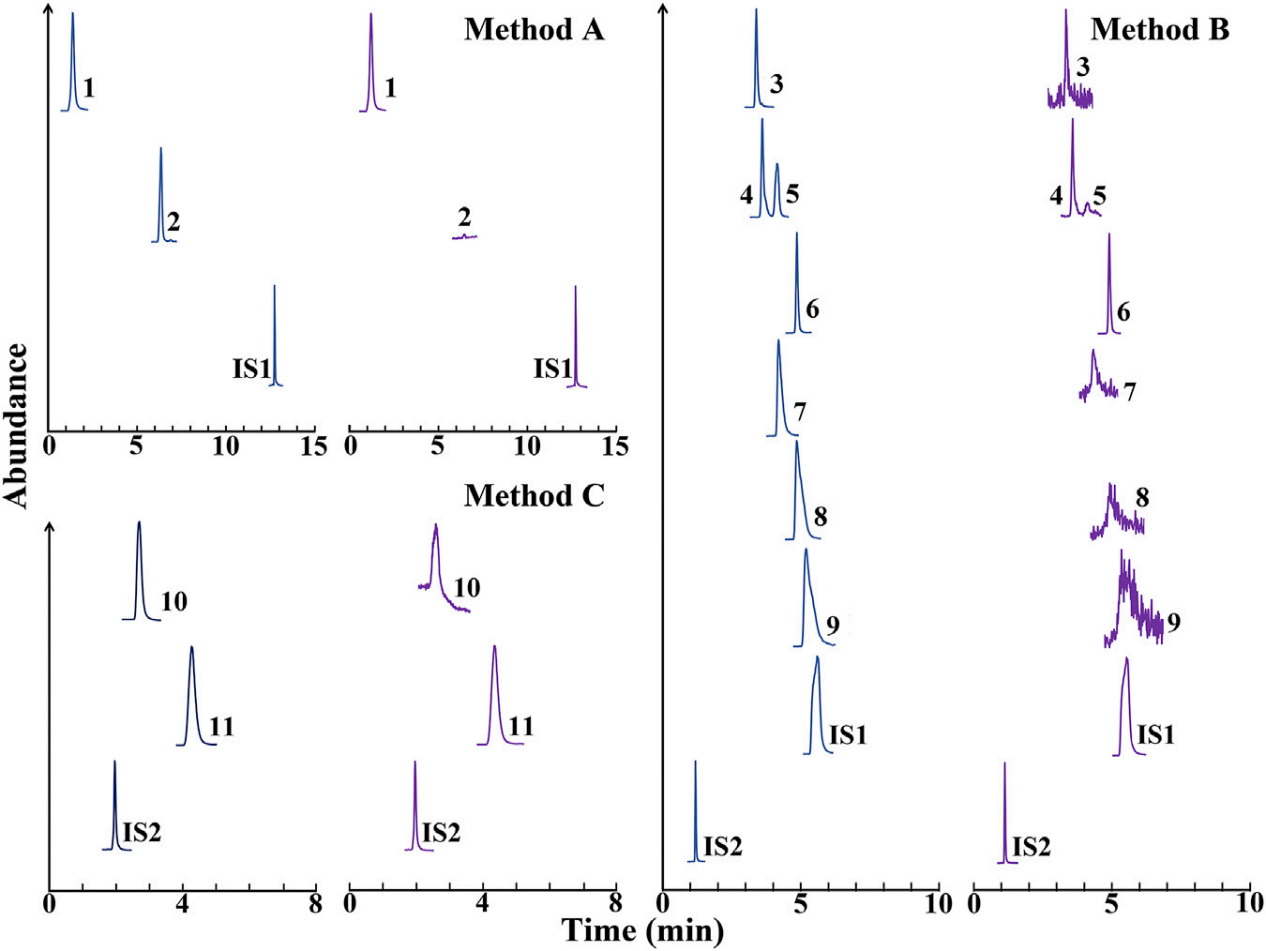
Representative multiple reaction monitoring chromatograms of the analytes in methods **(A,B,C)**. Blue peaks, reference compounds, and internal standards (IS); Purple peaks, *Coptidis Rhizoma* extract spiked with the reference internal standards. IS1, mycophenolic acid; IS2, tetraethylamine. 1, malic acid; 2, caffeic acid; 3, xylitol; 4, glucose; 5, fructose; 6, sucrose; 7, proline; 8, alanine; 9, glycine; 10, urea; 11, choline chloride.

The three methods were fully validated in terms of selectivity, sensitivity, linearity, intra-day, and inter-day variation, reproducibility, stability, and recovery. Representative MRM chromatograms of the analytes are shown in Figure 1. As shown in the chromatograms, no significant endogenous interference for the analytes and internal standards was observed in *Coptidis Rhizoma* extract. The calibration curves exhibited good linearity, and the corresponding typical regression equations are shown in Table 1. In addition, the lower limit of quantitation and lower limit of detection for the analytes indicated the high sensitivity of this method. Furthermore, the within-day and between-day precision (RSD%) of the analytes were all less than 4.86%, while the within-day and between-day accuracy were within 100 ± 5%. The methods for the analytes had good reproducibility (RSD < 4.77%), and the analytes were stable within 24 h at 4°C (RSD < 4.78%). The recovery was validated by spiking the reference analytes with a real *Coptidis Rhizoma* extract sample at three concentration levels. The recoveries of the analytes were between 88.41 and 110.14%, with RSD % between 0.03 and 5.16%. Taken together, these results suggest that the developed methods were specific, sensitive, reliable, and could be applied to quantitatively analyze the primary metabolites of *Coptidis Rhizoma* extract.

**TABLE 1:** Calibration curves, lower limit of quantitation (LLOQ), and lower limit of detection (LOD) of 11 analytes.

No	Analytes	Calibration curves	r	Linear range (ng/ml)	LLOQ (ng/ml)	LOD (ng/ml)
1	Malic acid	y = 0.0135 + 0.0049x	0.9974	3.91–500	3.91	1.96
2	Caffeic acid	y = −0.000138 + 0.00305x	0.9979	0.78–100	0.78	0.39
3	Xylitol	y = 0.00337 + 0.00092x	0.9966	15.63–1,000	15.63	7.81
4	Glucose	y = 0.01 + 0.000474x	0.9976	31.25–2000	31.25	7.81
5	Fructose	y = 0.0271 + 0.000692x	0.9973	31.25–2000	31.25	7.81
6	Sucrose	y = 0.325 + 0.0035x	0.9961	15.63–1,000	15.63	7.81
7	Proline	y = 0.0776 + 0.0345x	0.9989	3.13–200	3.13	1.56
8	Alanine	y = 0.115 + 0.0129x	0.9991	6.25–800	6.25	1.56
9	*Glycine*	y = 0.0172 + 0.00172x	0.9986	6.25–800	6.25	1.56
10	Urea	y = 3.7 + 0.189x	0.9966	6.25–200	6.25	3.13
11	Choline	y = 2.68 + 1.17x	0.9976	3.13–100	3.1	0.78

### Quantification of the Primary Metabolites in *Coptidis Rhizoma* Extract

Four primary metabolites, including malic acid, sucrose, glucose, and choline, were detected in nine batches of *Coptidis Rhizoma* extract. The levels of the primary metabolites are shown in Table 2. The result showed that in *Coptidis Rhizoma* extract, malic acid, glucose, sucrose, and choline chloride were the major primary metabolites, which had contents of 28.14, 16.10, 29.70, and 1.09 mg/g, respectively.

**TABLE 2:** Contents (mg/g) of four primary metabolites in nine batches of *Coptidis Rhizoma* extract (Mean ± SD, *n* = 3).

Batches	Malic acid	Glucose	Sucrose	Choline
1	27.17 ± 1.27	25.00 ± 1.11	39.47 ± 1.45	1.42 ± 0.04
2	27.00 ± 1.31	17.89 ± 0.71	36.73 ± 1.42	0.64 ± 0.02
3	28.27 ± 0.67	16.54 ± 0.73	22.93 ± 0.90	1.50 ± 0.06
4	38.70 ± 1.45	13.59 ± 0.39	42.47 ± 1.81	0.85 ± 0.03
5	25.57 ± 1.07	12.21 ± 0.19	22.33 ± 0.95	1.16 ± 0.03
6	29.83 ± 0.90	13.63 ± 0.27	28.47 ± 0.64	1.67 ± 0.03
7	19.67 ± 0.87	13.77 ± 0.29	14.77 ± 0.58	1.09 ± 0.06
8	24.93 ± 0.87	11.77 ± 0.55	26.60 ± 0.95	0.45 ± 0.01
9	32.13 ± 0.85	20.47 ± 0.46	33.53 ± 1.15	1.04 ± 0.02
Average	28.14 ± 5.26	16.10 ± 4.38	29.70 ± 9.06	1.09 ± 0.40

### Preparation, Stability, and Dissolving Capacity of the Prepared Natural Deep Eutectic Solvents

Several transparent and viscous NADESs (Supplementary Figure S1) were prepared in the following combinations: 1) malic acid and sucrose, 2) malic acid, sucrose, and choline chloride, 3) malic acid, sucrose, and glucose, and 4) malic acid, glucose, sucrose, and choline chloride. Given that the last combination included all the main small molecule primary metabolites in *Coptidis Rhizoma* extract, therefore it was used in subsequent studies.

**FIGURE 2:**
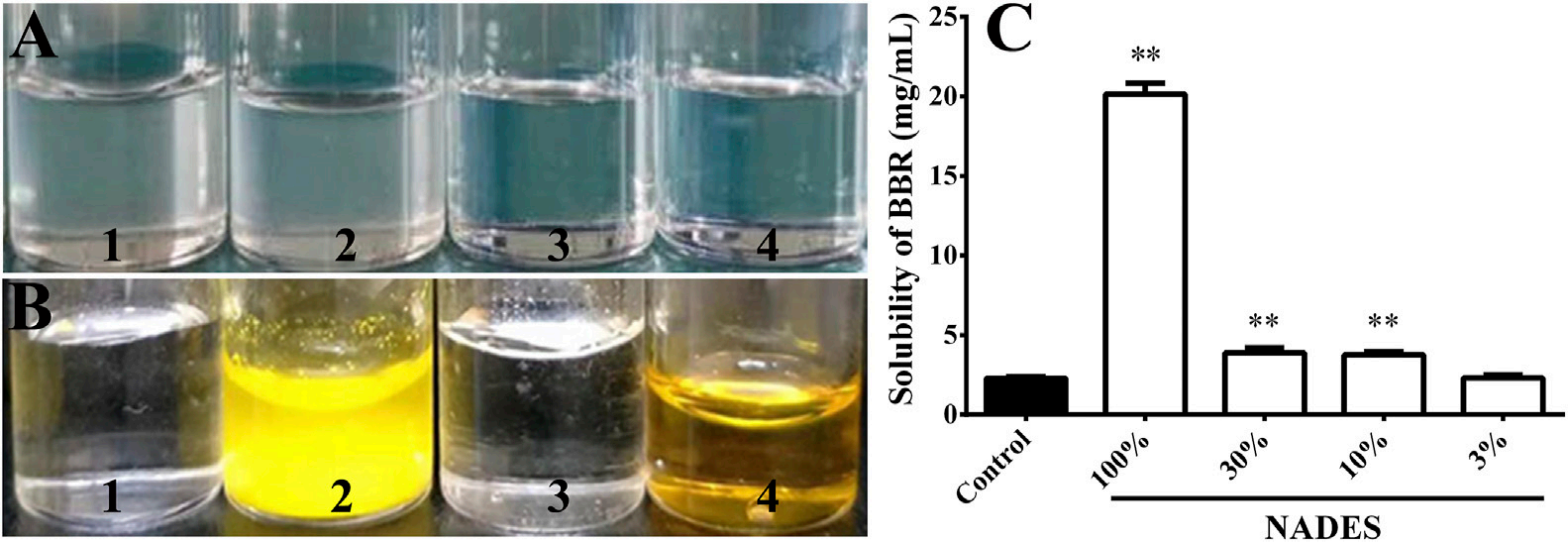
Stability and dissolving capacity of the prepared natural deep eutectic solvents (NADESs) (Mean ± SD, *n* = 4). **(A)**, the NADESs that were placed at room temperature (20°C) for 48 h after preparation. One to four, NADESs prepared by the addition of 250, 300, 350, or 400 *μ*L of water to 1,307.8 mg of the mixture of malic acid (490.5 mg), glucose (280.6 mg), sucrose (517.7 mg), and choline (19.0 mg), respectively. **(B)**, dissolving capacity of the NADES prepared with 350 *μ*L of water on berberine hydrochloride (BBR) at room temperature (20°C). One to four, water, BBR (20 mg/ml) dissolved in water, the NADES, and BBR (20 mg/ml) dissolved in the NADES, respectively. **(C)**, dissolving capacity of the NADES (100%) or its water dilutions (30, 10, and 3%) on berberine hydrochloride (BBR) at room temperature (20°C). **, *p* < 0.01 vs. control.

The level of water added to the formulations positively correlated with the stability of the formed NADESs. When more than 350 *μ*L of water was added to 1,307.8 mg of the mixture malic acid (490.5 mg), glucose (280.6 mg), sucrose (517.7 mg), and choline chloride (19.0 mg), the prepared NADESs were stable at room temperature for at least 48 h (Figure 2A). Therefore, the NADES containing 350 μL of water was used in subsequent studies. Based on the mass volume ratio, the density of the NADES was calculated as 1.32 ± 0.01 g/ml.

As shown in Figure 2B, the NADES significantly increased the solubility of BBR to at least 20 mg/ml (*p* < 0.01). The dissolving capacity of the NADES decreased sharply after dilution with water (Figure 2C). However, the solubility of BBR in 10 and 30% water dilutions of NADES was still higher (all *p* < 0.01) than that in water (Figure 2C).

### Acute Toxicity of the Natural Deep Eutectic Solvents and Its Dilutions in Mice

After oral administration of the NADES, the mice quickly became irritable and jumped around and then became dyskinesia and breathed rapidly. These mice all died within 6 h (Supplementary Figure S2). The autopsy revealed extensive necrosis of the liver, swelling of the stomach, and watery contents in the intestine (Supplementary Figure S3). However, after oral administration of 30% dilution of the NADES, only two mice died within 48 h (Supplementary Figure S2). In addition, the mice in 10% dilution of the NADES treatment group showed no obvious toxicity and no mice died after oral administration (Supplementary Figure S2). For this reason, in the follow-up pharmacokinetic and intestinal gut sac experiments, the highest concentration of NADES was limited at 10% (v/v).

### Cytotoxicity of the Natural Deep Eutectic Solvents and Its Dilutions in Madin-Darby Canine Kidney-Multidrug Resistance Gene-1 Cells

The results (Supplementary Figure S4) showed that the viability of the cells incubated with 3% dilution of the NADES for 4 and 24 h was completely lost (*p* < 0.01); the viability of the cells incubated with 1% dilution of the NADES was not influenced after incubation for 4 h but significantly decreased after incubation for 24 h (*p* < 0.01); while 0.3 and 0.1% dilutions of the NADES had no significant cytotoxicity after incubation for 4 and 24 h (*p* > 0.05). Therefore, 1% (v/v) was the highest concentration of the NADES in subsequent transportation experiment in cells and metabolic experiment in intestinal S9.

### Natural Deep Eutectic Solvents Dilutions Improved the Intestinal Absorption of Berberine Hydrochloride

**FIGURE 3:**
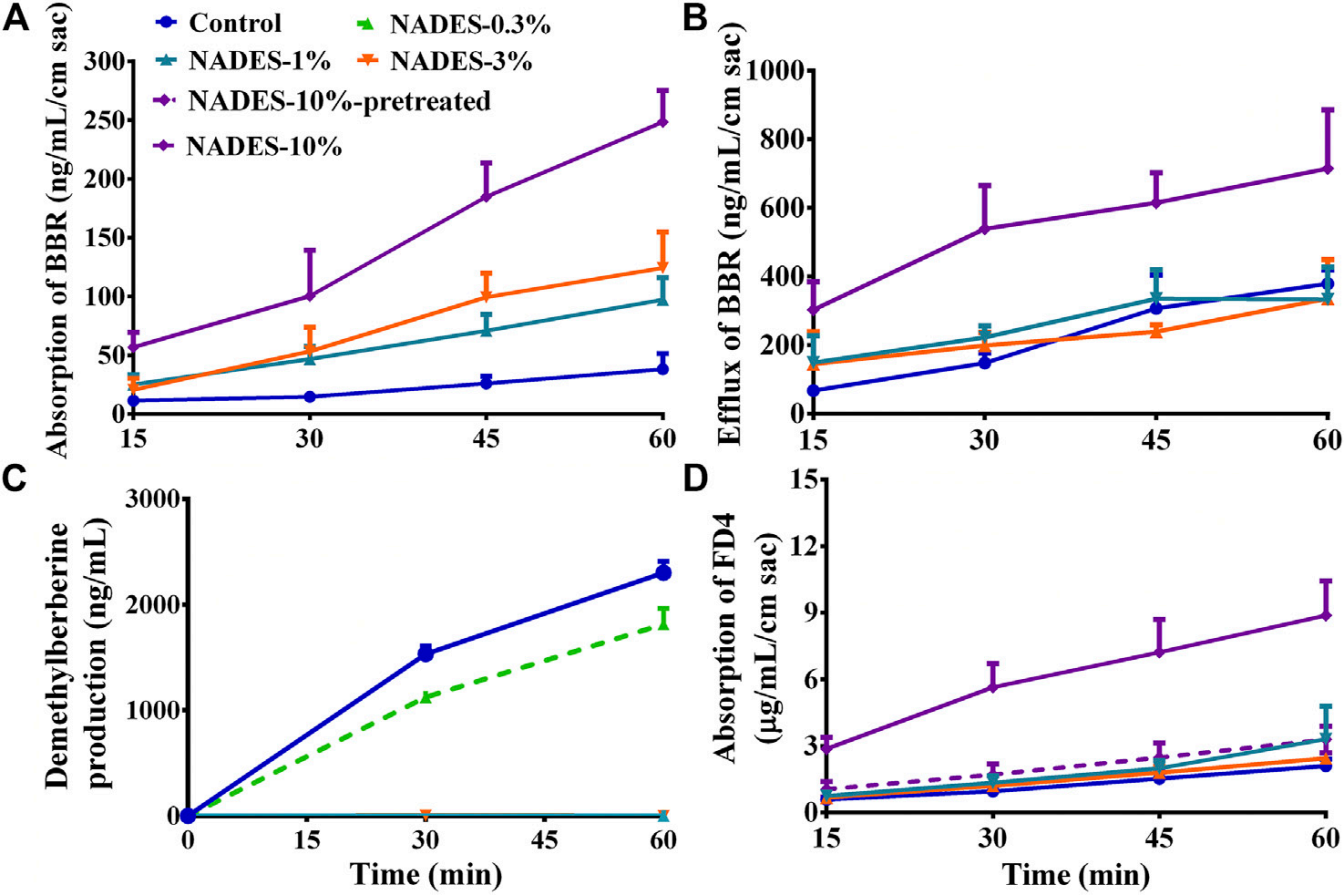
Studies in mouse gut sacs and intestinal S9 (Mean ± SD, *n* = 3). **(A,B)**, effects of the dilutions (1, 3, and 10%) of the natural deep eutectic solvent (NADES) on the absorption and efflux of berberine hydrochloride (BBR, 200 *μ*g/ml) in mouse gut sacs, respectively; **(C)**, effects of the dilutions (0.3 and 1%) of the NADES on the metabolism of BBR (10 *μ*g/ml) in intestinal S9; **(D)**, effects of the diluted NADES (1, 3, and 10%) and pretreatment with 10% NADES for 4 h on the transportation of fluorescein isothiocyanate-labeled dextran (FD4, 3 mg/ml) in mouse gut sacs.

In the *in vitro* transportation experiment with mouse gut sacs, NADES dilutions (1–10%) increased (*p* < 0.01) the transportation of BBR from the mucosal side to the serosal side in a concentration-dependent manner (Figure 3A). Surprisingly, NADES dilutions also increased (*p* < 0.01) the transportation of BBR from the serosal side to the mucosal side when its concentration was higher than 10% (Figure 3B). The results (Table 3) of *in vitro* experiments carried out with MDCK-MDR1 cells showed that the *P*_*app*_ from AP to BL of BBR was lower than 10^−6^ cm/s, indicating that its permeability was poor. In addition, the ER of BBR was as high as 32.3, which is far greater than the critical value of two (Giacomini et al., 2010), indicating that P-gp-mediated efflux significantly restricted BBR uptake. The NADES dilutions significantly decreased the ER of BBR (Table 3). Consistent with the results obtained from the gut sac experiment, the NADES increased both the *P*_*app*_ from BL to AP and the *P*_*app*_ from AP to BL. The above results indicated that the mechanism by which NADES promoted BBR intestinal absorption was not due to the inhibition of P-gp-mediated efflux.

**TABLE 3:** Influences of the natural deep eutectic solvent (NADES) on the apparent permeability (*P*_*app*_) and efflux rates (ERs) of berberine hydrochloride in MDCK-MDR1 cells (Mean ± SD, *n* = 3).

Groups	*P*_*app (AP-BL)*_ (×10^−6 ^cm/s)	*P*_*app (BL-AP)*_ (×10^−6 ^cm/s)	ERs
Control	0.41 ± 0.06	13.3 ± 0.4	32.3
0.3% NADES	0.88 ± 0.13	13.1 ± 1.9	14.9*
1% NADES	20.1 ± 8.7	21.7 ± 1.4	1.1**

** *, *p* < 0.05; , *p* < 0.01 vs. Control.

The results of *in vitro* metabolism experiments showed that the production of demethylberberine, a representative metabolite of BBR, was completely inhibited (all *p* < 0.01) by the NADES dilutions when its concentration was higher than 1% (Figure 3C), suggesting that the NADES dilutions increased the intestinal metabolic stability of BBR in a concentration-dependent manner.

In addition, when the concentration of the NADES dilutions reached 10%, it increased (*p* < 0.01) the absorption of FD4 across mouse gut sacs (Figure 3D), suggesting that the NADES dilutions could open the tight junctions between enterocytes. Importantly, compared with the 10% NADES-treated group, the effect of 10% NADES dilutions pretreatment on FD4 absorption was negligible (*p* < 0.01) (Figure 3D), which suggests that the influence of the NADES dilutions on tight junctions was reversible.

### Natural Deep Eutectic Solvents Dilutions Improved the Pharmacokinetics of Berberine Hydrochloride in Mice

**FIGURE 4:**
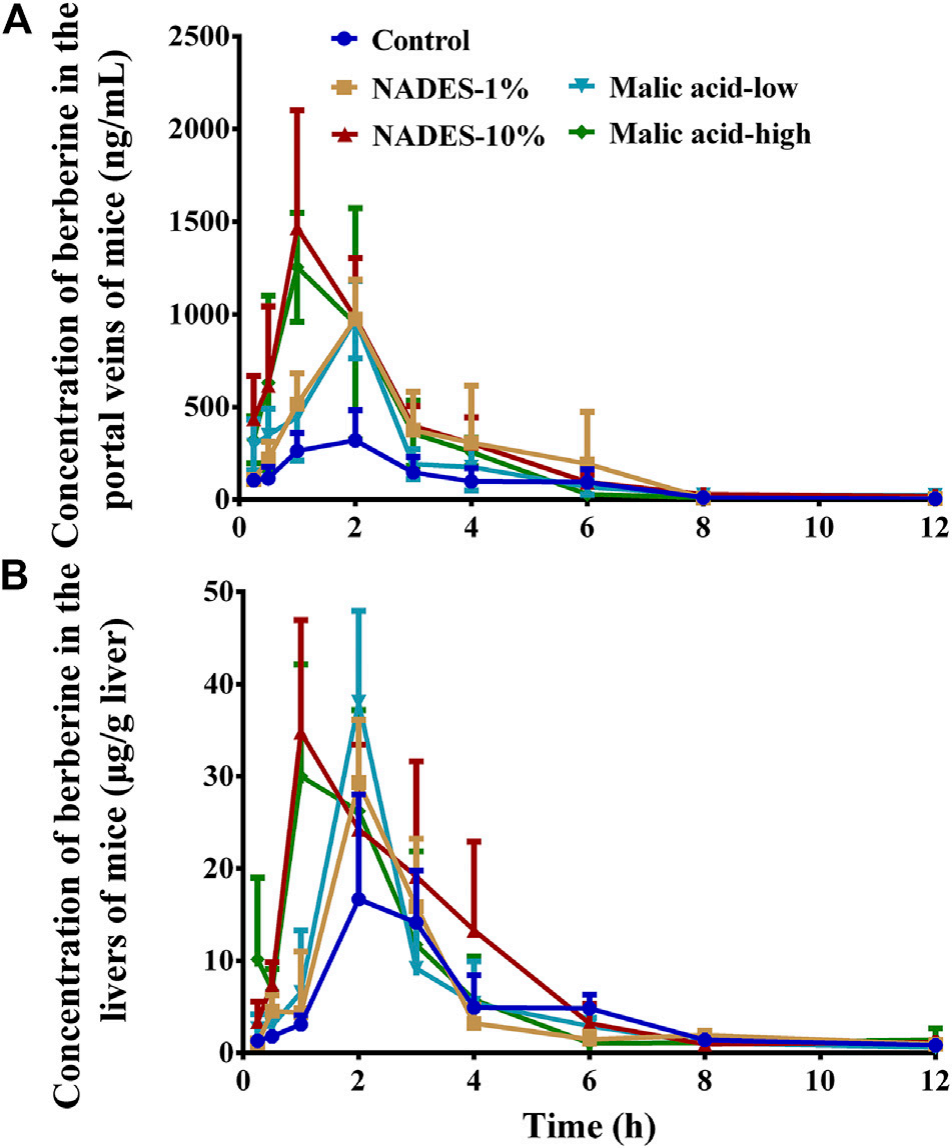
Concentration time-curves of berberine in the portal veins **(A)** and livers **(B)** of mice that were orally administered with berberine hydrochloride (BBR, 200 mg/kg) (Mean ± SD, *n* = 6). BBR was dissolved in water (control), water dilutions (1 and 10%) of the natural deep eutectic solvent (NADES), or water solutions of malic acid (low, 98 mg/kg; high, 980 mg/kg), respectively.

As shown in Figure 4 and Table 4, the NADES dilutions concentration dependently improved the pharmacokinetic properties of BBR in mice receiving oral BBR. Decreased T_max_ and increased C_max_ and AUC_0–12 h_ of BBR in the portal vein indicated that NADES dilutions promoted the intestinal absorption of BBR. In the portal vein, the C_max_ of BBR in the 1 and 10% dilutions of NADES treated group was 3.04 and 4.57 times of that in the control group, while the AUC_0–12 h_ was 2.45 and 3.22 times, respectively. Furthermore, the pharmacokinetic parameters of BBR were improved in the liver, one of the major target organs. In the liver, the C_max_ of BBR in the 1 and 10% dilutions of NADES treated group was 1.75 and 2.08 times of that in the control group, while the AUC_0–12 h_ was 1.12and 1.65 times, respectively.

**TABLE 4:** Pharmacokinetic parameters of berberine in the portal veins and livers of the mice that were orally administered with berberine hydrochloride (BBR, 200 mg/kg) (Mean ± SD, *n* = 6).

Samples	Parameters	Control	NADES (%)		Malic acid (mg/kg)	
			1	10	98	980
Portal vein	T_1/2_ (h)	1.7	1.3	1.7	2.7	1.4
	T_max_ (h)	2.0	2.0	1.0	2.0	1.0
	C_max_ (ng/ml)	320.6	974.7	1465.8	972.3	1254.7
	AUC_0–12 h_ (ng·h/mL)	1115.1	2733.0	3587.9	2230.5	3058.3
	AUC_0-∞_ (ng·h/mL)	1126.4	2740.4	3614.7	2296.0	3065.9
	MRT (h)	3.1	2.9	2.4	2.7	2.1
Livers	T_1/2_ (h)	2.2	4.5	2.2	2.3	1.5
	T_max_ (h)	2.0	2.0	1.0	2.0	1.0
	C_max_ (μg/g liver)	16.7	29.3	34.8	38.0	30.0
	AUC_0–12 h_ (μg·h/g liver)	66.3	74.5	109.4	72.3	85.9
	AUC_0-∞_ (μg·h/g liver)	68.4	80.9	112.5	73.8	88.3
	MRT (h)	3.7	3.2	3.0	3.0	2.5

BBR was dissolved in water, dilutions of the NADES (1 and 10%), or solutions of malic acid (98 or 980 mg/kg, respectively equal to the dosage of malic acid in 1% or 10% NADES dilution), respectively. AUC, the area under the concentration time curve; C_max_, peak concentration; MRT, mean retention time; T_1/2_, elimination half-life; T_max_, time to reach peak concentration.

### Role of Malic Acid in the Natural Deep Eutectic Solvents

**FIGURE 5:**
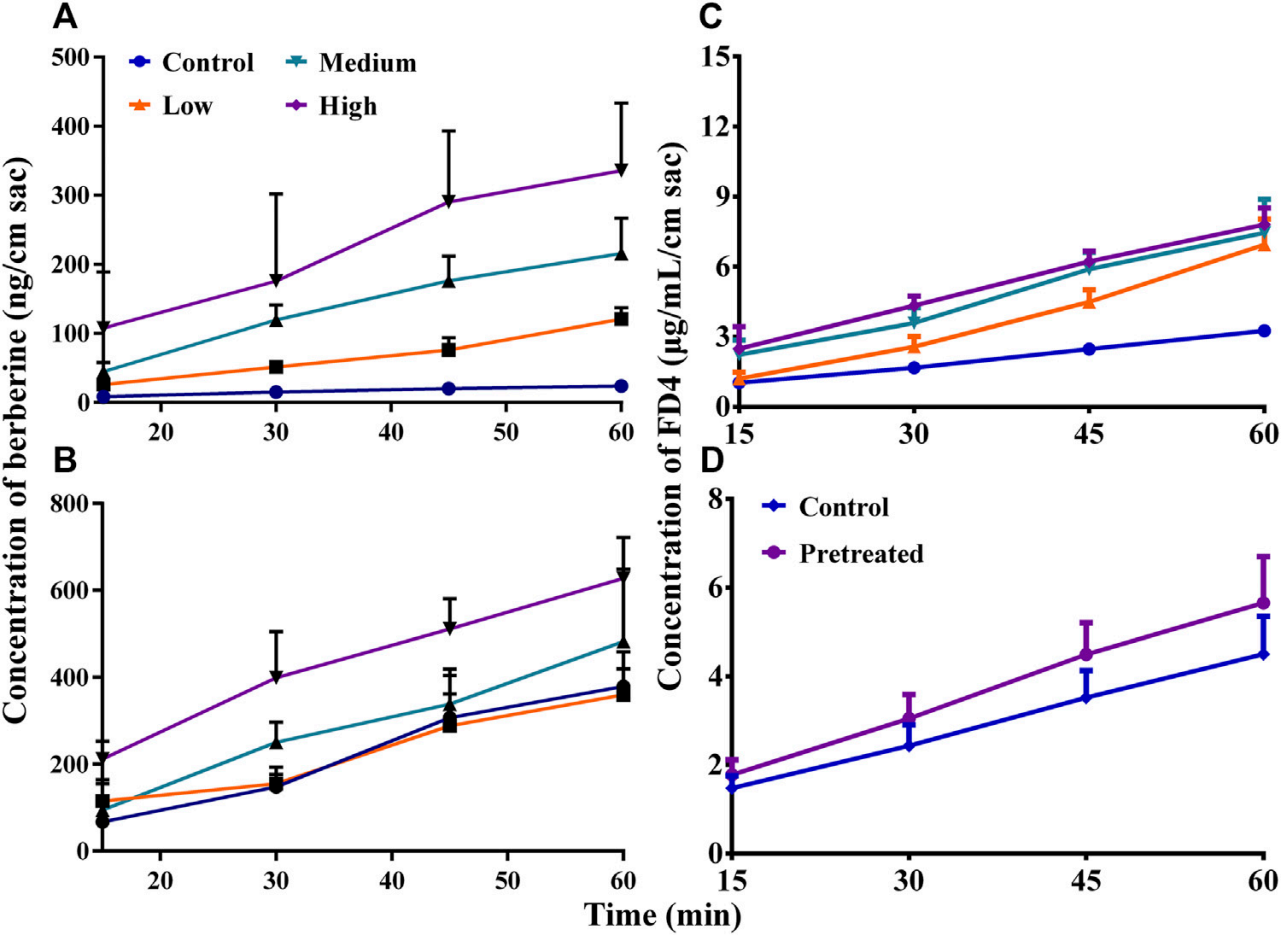
Studies in mouse gut sacs (Mean ± SD, *n* = 3). **(A,B)**, effects of malic acid on the absorption and efflux of berberine hydrochloride (BBR, 200 *μ*g/ml) in mouse gut sacs, respectively; **(C,D)**, effects of the presence and pretreatment (for 4 h) of malic acid on the absorption of fluorescein isothiocyanate-labeled dextran (FD4, 3 mg/ml) in mouse gut sacs, respectively. Low, medium, and high: 4.9, 14.7, or 49.0 mg/ml, respectively.

Among the four small molecule primary metabolites that make up the NADES, only pure malic acid improved the solubility of BBR (Supplementary Figure S5). When the concentration of malic acid was higher than 40 mg/ml, the solubility of BBR was significantly increased (*p* < 0.01) (Supplementary Figure S6). Interestingly, the solubility of berberine hydrochloride was increased as the pH of the malic acid solution was increased by the addition of NaOH (*p* < 0.01) (Supplementary Figure S7). The results indicated that malic acid improves the solubility of berberine hydrochloride not because of its acidity. Malic acid also showed significant acute toxicity in mice. The number and time of death of mice in each group were similar to the NADES and corresponding dilutions (Supplementary Figures S8, S9). In addition, the viability of MDCK-MDR1 cells was completely lost after incubation with 4.9 mg/ml malic acid for 4 and 24 h (*p* < 0.01), but no significant cytotoxicity was observed after incubation with 1.47 or 0.49 mg/ml malic acid for 4 and 24 h (*p* > 0.05) (Supplementary Figure S10). In gut sacs, malic acid promoted the absorption of BBR in a concentration-dependent manner in the range of 4.9–49.0 mg/ml (Figure 5A); it also promoted the efflux of BBR when its concentration reached 14.7 mg/ml or more (Figure 5B). Malic acid concentration dependently and increased the absorption of FD4 across mouse gut sacs (all *p* < 0.01) (Figure 5C), but the effect was reversible (Figure 5D). Finally, as shown in Figure 4 and Table 4, malic acid concentration dependently improved the pharmacokinetic properties of oral BBR in mice receiving oral BBR.

## Discussion and Conclusion

In this study, three LC-MS/MS-based methods were first established and fully validated to determine the levels of 11 primary metabolites of *Coptidis Rhizoma* extract. These metabolites were selected because they were usually used for artificial preparation of NADESs. The methods were simple, reliable, and suitable for quantitative analysis. The results showed that sucrose, malic acid, glucose, and choline are the four major primary metabolites in *Coptidis rhizoma* extract.

NADESs can be artificially prepared using several methods, including the classic vacuum evaporation and heating methods (Dai et al., 2013). In this study, NADESs were prepared using the heating method, which suggests that NADESs could be concomitantly formed during the preparation of *Coptidis Rhizoma* extract. In other words, NADESs would be “endogenously” present in the *Coptidis Rhizoma* extract. The most stable stoichiometric proportion of HBA and HBD is usually 1:1 (Choi et al., 2011; Dai et al., 2013). However, it has also been reported that it is not necessary for NADESs to have a fixed stoichiometric proportion of HBA and HBD (Martins et al., 2019). In this study, a stable NADES was prepared according to the content proportion of the four primary metabolites in the *Coptidis Rhizoma* extract. As far as we know, this is the first study to report the use of several primary metabolites in a plant extract for the preparation of NADESs based on the ratio of their contents in the extract. Consistent with previous reports (Choi et al., 2011; Dai et al., 2015), water was essential for the formation of the “endogenous” NADES. In addition, the water content positively correlated with the stability of the NADES, that is, within a certain range, the higher the water content, the more stable the NADES. This is possibly because water molecules may act as linkers between the components of NADESs (Liu et al., 2018b).

It has been reported that about 56% of BBR is directly discharged from the body after oral administration, which is related to the poor solubility of BBR (Liu et al., 2016). The common dosage of BBR in mice is 200 mg/kg (Liu et al., 2016); therefore, if BBR is administered at 0.2 ml/10 g body weight, its concentration should be 10 mg/ml. In this study, the NADES could dissolve at least 20 mg/ml BBR, suggesting the strong capability of the NADES to dissolve BBR. It has been reported that a large amount of water would destroy the structure of NADESs (Dai et al., 2013; Dai et al., 2015; Hammond et al., 2017), explaining why the dissolving capacity of the NADES dropped sharply after dilution with water. Interestingly, after dilution with 10 × water, the NADES dilution retained a significant ability to dissolve BBR.

The effect of NADESs on the oral bioavailability of compounds was first reported for rutin in 2016 (Faggian et al., 2016). The effect of artificially prepared NADESs on the oral bioavailability of BBR was first reported in 2017 (Sut et al., 2017). It has been reported that the effects of NADESs on the pharmacokinetics of other compounds is predominantly dependent on their solubilizing mechanisms (Chen et al., 2017). However, for the first time, other pharmacokinetic mechanisms were explored in this study and discussed below.

Cytochrome P450 enzymes (CYPs), especially CYP3A, play a major role in the phase I metabolism of BBR (Liu et al., 2009). There are a variety of CYPs in the intestinal epithelial cells, especially CYP3A, which is highly expressed in the intestine (Paine et al., 2006). Moreover, there are many efflux transporters, such as P-gp, in the intestinal epithelial cells, which significantly reduce the uptake of their substrates. BBR is a substrate of both CYP3A4 and P-gp, which explains why 43.5% of oral BBR is eliminated during intestinal absorption (Liu et al., 2016). Although the detailed mechanism was unclear, the results in this study showed that the NADES dilutions strongly inhibited the intestinal metabolism of BBR. The results obtained in the gut sacs and MDCK-MDR1 cell-based studies suggested that the NADES dilutions reversed P-gp-mediated BBR efflux. However, given that high concentrations of the NADES dilutions (10% in the gut sacs and ˃1% in the MDCK-MDR1 cells) also promoted the efflux of BBR, other mechanisms besides the inhibition of the function of P-gp were supposed to be involved. It has been reported that NADESs induce the production of reactive oxygen species and increase the release of lactate dehydrogenase by damaging the cell membrane (Hayyan et al., 2015; Mbous et al., 2017). Therefore, NADESs may be able to modify the permeability of the cell membrane of enterocytes. Furthermore, the results obtained from an FD4 study suggested that at a high concentration, NADESs could act as a chemical permeation enhancer (CPE) (Deli, 2009) to reversibly open the tight junctions of enterocytes. Interestingly, CPEs such as sodium caprate, an anionic surfactant, and chitosan, a cationic polysaccharide, have been used to increase the pharmacokinetics of oral BBR (Liu et al., 2016). In addition, some natural products, including small-molecule constituents of herbal extracts, such as saponins (Narai et al., 1997) and alkaloids (Lu et al., 2010; Li et al., 2013; Watari et al., 2015) have been reported as CPEs. CPEs may act directly and specifically on the extracellular loops of tight junction proteins and TJ-associated membrane microdomains (Deli, 2009). Further studies are required to fully elucidate the detailed mechanism of action of the NADES dilutions.

The extremely low oral bioavailability limits the further research and development of BBR (Liu et al., 2016). Therefore, various strategies have been used to improve the oral bioavailability of BBR (Liu et al., 2016). The pharmacokinetic study showed that the NADES dilutions mainly affected the absorption process of oral BBR. The NADES dilutions promoted the intestinal absorption of BBR *in vivo* in a concentration-dependent manner, which was manifested by a significant increase in BBR exposure levels (AUC_0–t_ and C_max_) in the portal vein of the mice. The level of BBR distributed in the liver increased mainly due to increased intestinal absorption. The influence of the NADES dilutions on the pharmacokinetics of oral BBR in systemic circulation was not investigated due to the poor dose-exposure relationship of BBR in systemic circulation (Li et al., 2018). The increase in intestinal absorption of oral BBR and its *in vivo* exposure levels are undoubtedly beneficial for improving its systemic effects. However, it should be noted that BBR itself has acute toxicity (Ma et al., 2010), with a mechanism related to cholinesterase inhibition (Ma et al., 2011). Therefore, in order to avoid toxic reaction, the *in vivo* exposure level of BBR should be controlled within a certain range. Researches on the relationship between dose-exposure level-toxicity are expected to achieve a balance of benefits and risks. In this experiment, no toxic reaction was found after oral administration of BBR that was dissolved in the NADES dilutions.

It has been reported that there are large pharmacokinetic differences between BBR in *Coptidis Rhizoma* extract and pure BBR (Ma et al., 2016a). The results obtained in this study suggested that the “endogenous” NADES might contribute to the improved pharmacokinetics of BBR in *Coptidis Rhizoma* extract. In addition, it was found that BBR in *Coptidis Rhizoma* extract had a non-linear dose exposure relationship after oral administration (Li et al., 2018). That is, when the dose of *Coptidis Rhizoma* extract reached 3 g/kg, the *in vivo* exposure level of BBR was much higher than the exposure level that was predicated according to dose proportion relationship (Li et al., 2018). It is worth noting that the concentration of 4.9 mg/ml in this study is close to the concentration of malic acid (about 4.2 mg/ml) when *Coptidis Rhizoma* extract is orally administered at a dosage of 3 g/kg and a volume of 20 ml/kg. The results suggested that malic acid and the malic acid based NADES may contribute to the non-linear dose exposure relationship of BBR in oral *Coptidis Rhizoma* extract.

It is undeniable that the NADES has shortcomings as a CPE for oral BBR. Above all, the NADES itself showed acute toxicity to mice. It has been reported that NADESs may block and halt blood flow due to high intrinsic viscosity (Mbous et al., 2017). For this reason, only the NADES dilutions, which showed no obvious acute toxicity in mice, instead of the NADES itself, was used in subsequent study. In addition, the chronic toxicity of the NADES dilutions was unclear. Moreover, the NADES dilutions contained glucose and sucrose, which may influence the blood glucose of experimental animals. In the future research, the component and their ratios of the NADES should be optimized to address such concerns. However, the NADES dilutions could be used to improve the pharmacological effects of BBR that are not closely related to glucose and lipid metabolism.

After being diluted, the NADES can no longer be regarded as a NADES. However, the NADES dilutions cannot be regarded as an aqueous solution of the four primary metabolites, because they may retain weak intermolecular interactions (Supplementary Figure S11). The residual intermolecular interactions may contribute to the physicochemical and biological properties of the NADES dilutions. Therefore, it was expressed as “NADES dilution” instead of “NADES” or “solution of the primary metabolites” in the manuscript.

Malic acid was identified as a major component in the NADES in terms of solubility, acute toxicity, cytotoxicity, and influences on the intestinal absorption and pharmacokinetics of oral BBR. The results are consistent with the literature reports, that is, the physicochemical and biological properties of NADESs, such as solubility (Sut et al., 2017), cytotoxicity (Paiva et al., 2014; Radosevic et al., 2015), are closely related to the properties of its components. It should be noted that compared with pure malic acid, the corresponding NADES dilutions had lower cytotoxicity, stronger dissolving capacity, and better pharmacokinetic-improving effects on oral BBR. For example, in the portal vein of the mice in 1 and 10% NADES dilutions treated group, the C_max_ of BBR was, respectively, 3.04 and 4.57 times and the AUC_0–12 h_ was, respectively, 2.45 and 3.22 times of that in the control group. In contrast, in the 98 mg/kg and 980 mg/kg malic acid treated groups, the C_max_ was, respectively, 3.03 and 3.91 times while the AUC_0–12 h_ was, respectively, 2.00 and 2.74 times of that in the control group.

In summary, a stable NADES was prepared according to the content proportion of the four primary metabolites in *Coptidis Rhizoma* extract. The dilutions of the NADES promoted the intestinal absorption of oral BBR by increasing solubility, reducing intestinal efflux, improving intestinal metabolic stability, and reversibly opening the tight junctions between enterocytes. This study showed for the first time that “endogenous” NADESs could be prepared from herbal extracts and could improve the pharmacokinetic properties of coexisting active constituents. This study demonstrated the pharmacokinetic synergy of the constituents of *Coptidis Rhizoma* extract. In addition, this study was the first to systematically show the mechanisms through which NADESs improve the pharmacokinetics of another compound, which may help to promote the use of NADESs in oral drug delivery.

## Data Availability

The original contributions presented in the study are included in the article/Supplementary Material, further inquiries can be directed to the corresponding authors.

## Abbreviations

BBR, berberine hydrochloride; CPE, chemical permeation enhancer; CYPs, cytochrome P450 enzymes; DES, deep eutectic solvent; DMEM, Dulbecco’s modified Eagle’s medium; ER, efflux rate; ESI, electrospray ionization; FD4, fluorescein isothiocyanate-labeled dextran with a molecular weight of 4,400 g/mol; HBA, hydrogen bond acceptor; HBD, hydrogen bond donor; HBSS, Hank’s balanced salt solution; LC-MS, liquid chromatography-mass spectrometry; LC-MS/MS, liquid chromatography tandem mass spectrometry; MDCK-MDR1, Madin-Darby canine kidney cells stably expressing the transporter p-glycoprotein; NADES, natural deep eutectic solvent; Papp, permeability; P-gp, p-glycoprotein.
