# Supplementary material for: Intra-Herb Interactions: Primary Metabolites in Coptidis Rhizoma Extract Improved the Pharmacokinetics of Oral Berberine Hydrochloride in Mice
Source: Front Pharmacol. 2021 Jun 7;12:675368. doi: 10.3389/fphar.2021.675368 (PMC8215677; doi:10.3389/fphar.2021.675368)
Supplement: Supplementary file 1 [file DataSheet2.PDF]

NMR analysis

D_2O (Cambridge isotope laboratories, Massachusetts, USA) was used to prepare the solutions of malic acid, glucose, sucrose, and choline chloride. In addition, D_2O was used to prepare the NADES and its 30%, 10%, 3%, 1%, 0.3% and 0.1% dilutions. The solutions were then transferred to test tubes and analyzed at 25 ℃ by using a Bruker Avance Neo 600 MHZ NMR spectrometer (Massachusetts, USA).

It was found (Fig. S11) that there was no hydrogen peak showing coupling or splitting information in the NADES, which might be due to poor shimming caused by high viscosity of the NADES. However, the chemical shifts of each compound in the dilutions of NADES (30%, 10%, 3%, 1%, 0.3%, and 0.1%) were significantly different from corresponding pure compound. For example, compared with pure malic acid, the proton signals of malic acid in the dilutions of NADES move to the low field region.
