# Supplementary figures and images for: Intra-Herb Interactions: Primary Metabolites in Coptidis Rhizoma Extract Improved the Pharmacokinetics of Oral Berberine Hydrochloride in Mice
Source: Front Pharmacol. 2021 Jun 7;12:675368. doi: 10.3389/fphar.2021.675368 (PMC8215677; doi:10.3389/fphar.2021.675368)

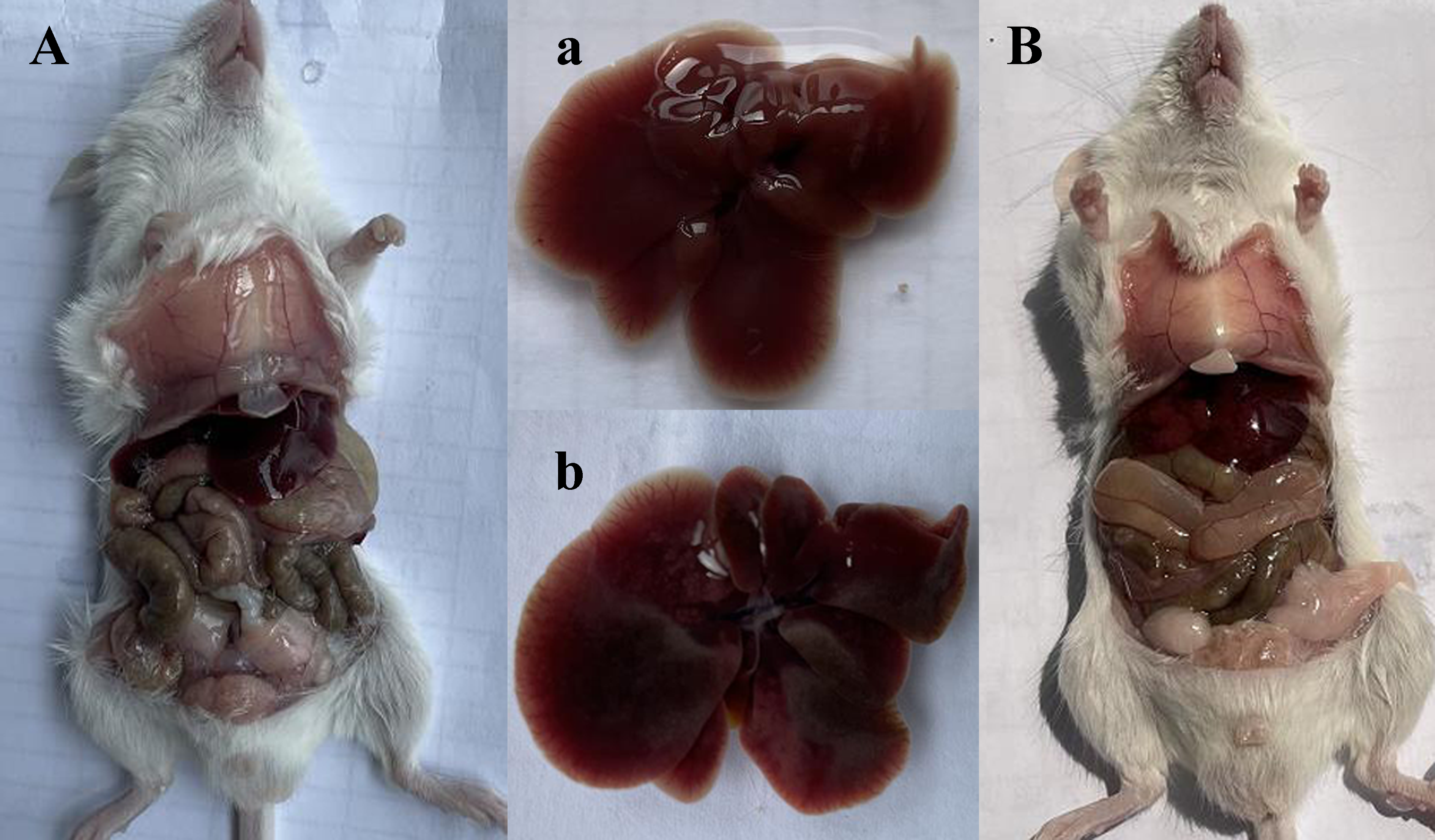

Supplement: Supplementary file 3 [file Image3.TIF]

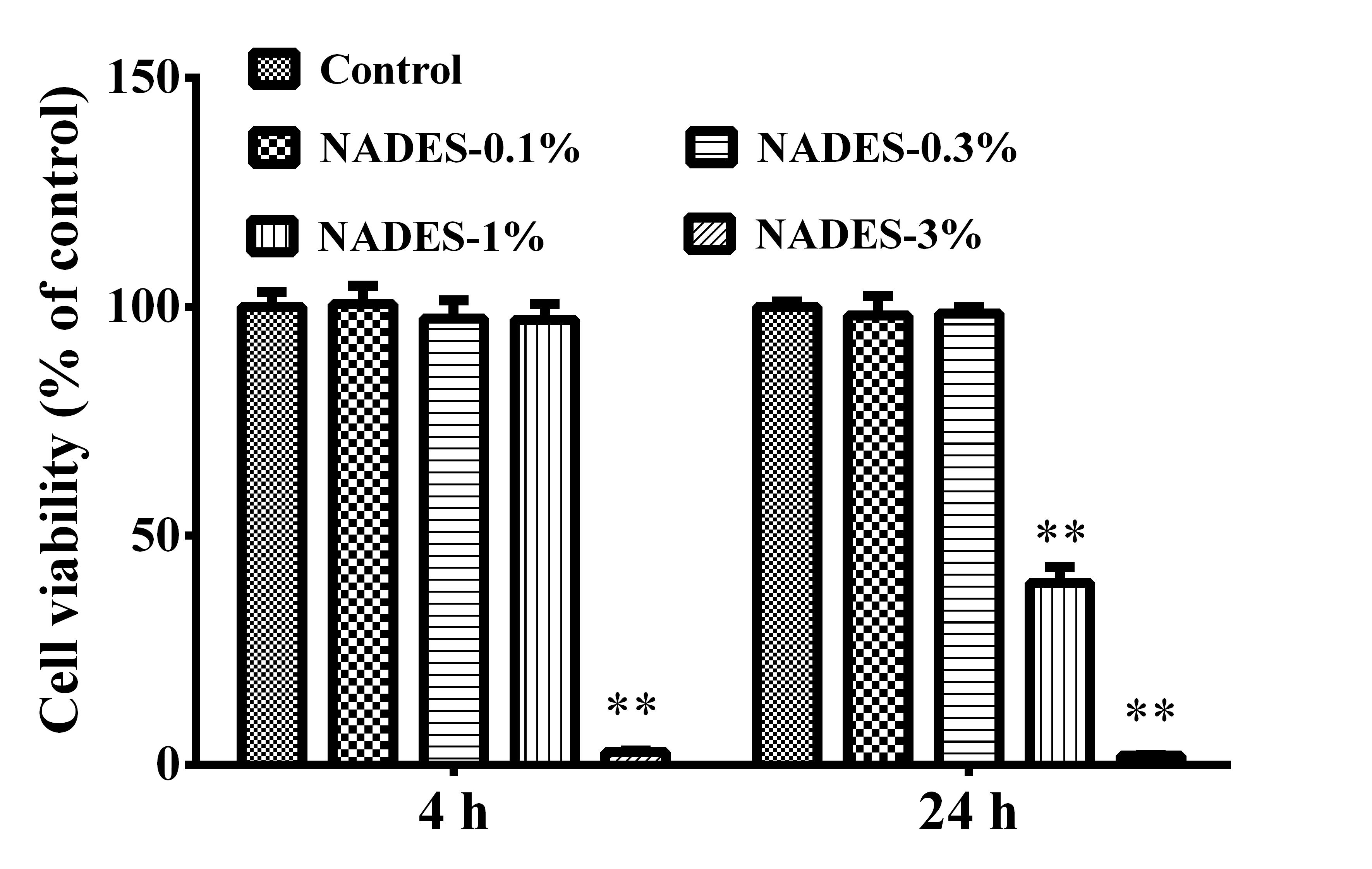

Supplement: Supplementary file 4 [file Image4.TIF]

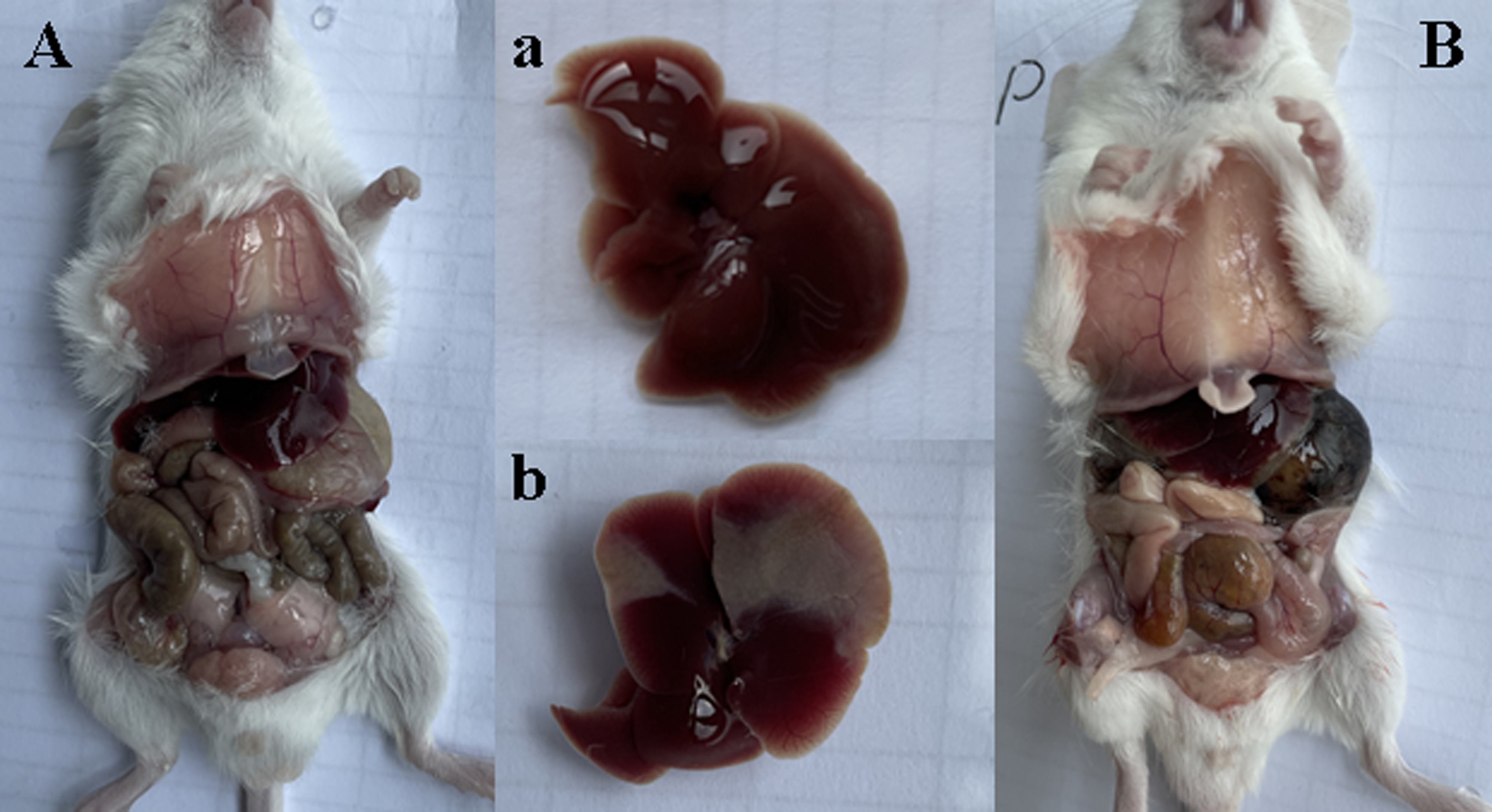

Supplement: Supplementary file 5 [file Image9.TIF]

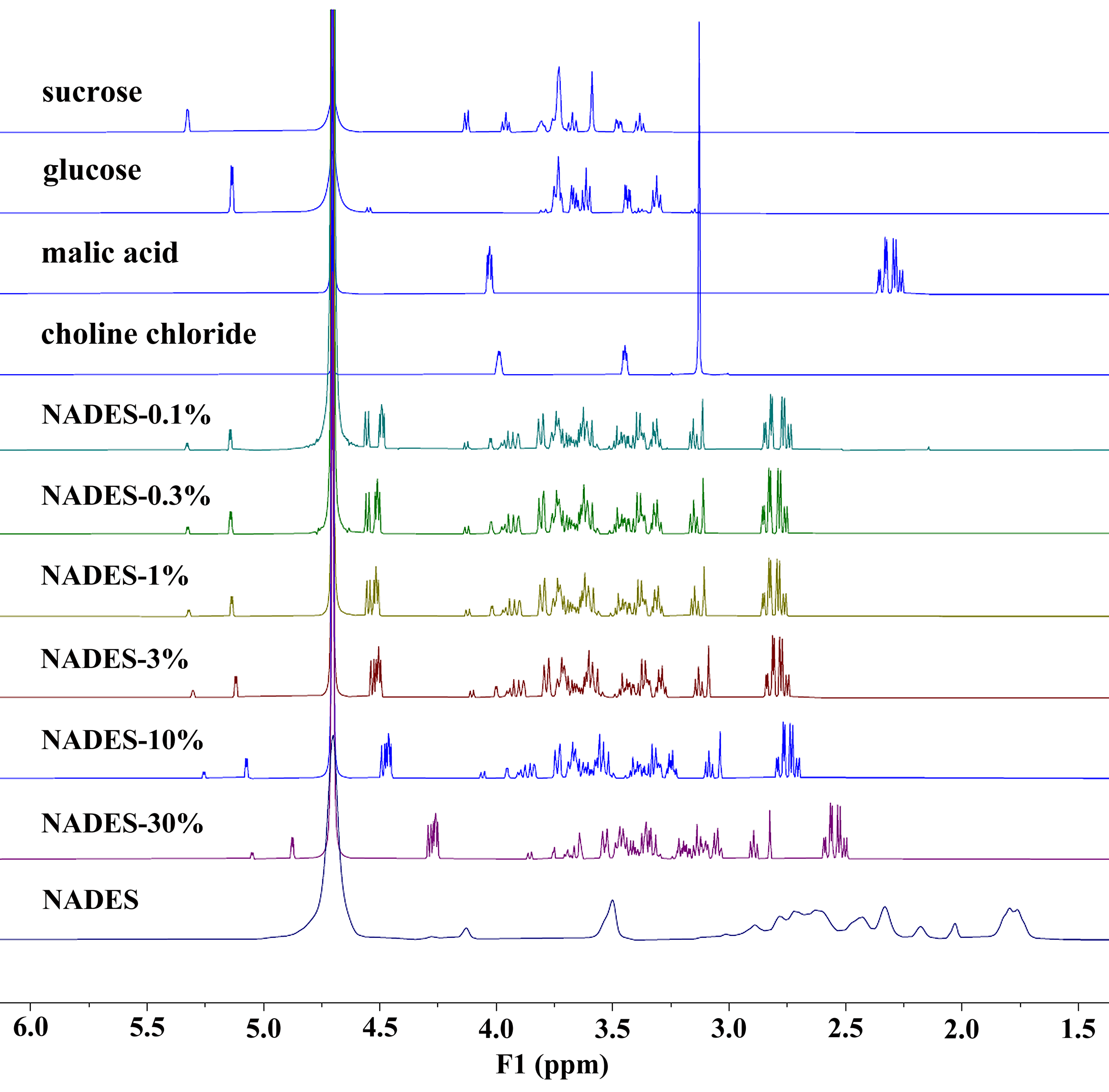

Supplement: Supplementary file 7 [file Image11.TIF]

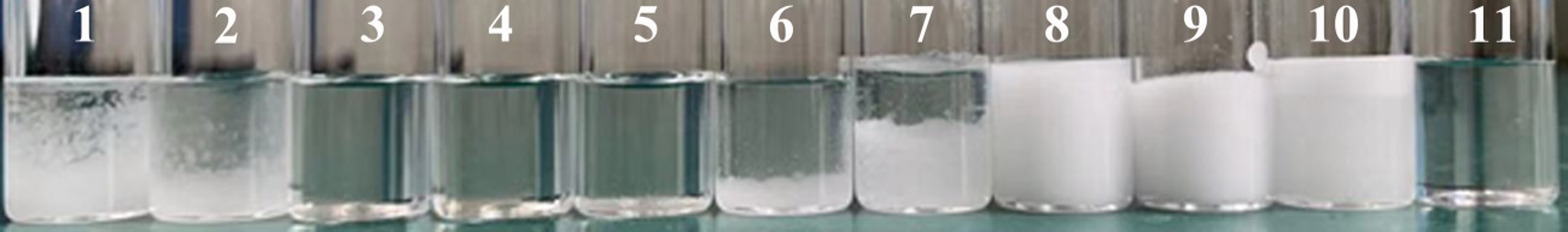

Supplement: Supplementary file 8 [file Image1.TIF]
