# Supplementary material for: Intra-Herb Interactions: Primary Metabolites in Coptidis Rhizoma Extract Improved the Pharmacokinetics of Oral Berberine Hydrochloride in Mice
Source: Front Pharmacol. 2021 Jun 7;12:675368. doi: 10.3389/fphar.2021.675368 (PMC8215677; doi:10.3389/fphar.2021.675368)
Supplement: Supplementary file 11 [file DataSheet1.PDF]

Figure legends

Fig.S1. Natural deep eutectic solvents (NADESs) prepared based on different combinations of four major small molecule primary metabolites in *Coptidis Rhizoma* extract. About 490.5 mg of malic acid, 280.6 mg of glucose, 517.7 mg of sucrose, and 19.0 mg of choline chloride were mixed together according to different combinations, then 350 μL of water was added to prepare NADESs by the heating method. The prepared NADESs were then placed at about 20 ℃ for 48 hours after preparation. 1, malic acid + glucose + choline chloride; 2, sucrose + glucose + choline chloride; 3, malic acid +sucrose + choline chloride; 4, malic acid+ sucrose + glucose; 5, malic acid + sucrose; 6, sucrose + glucose; 7, sucrose + choline chloride; 8, malic acid + glucose; 9, malic acid + choline chloride; 10, glucose + choline chloride; 11, malic acid+ glucose + sucrose + choline chloride.

Fig.S2. Survival curves of mice that were orally administered with the natural deep eutectic solvent (NADES) or its dilutions (n = 20). The mice were orally administered with 0.2 ml/10 g body weight the NADES or its water dilutions (30% and 10%), respectively. Then the death time of mice were recorded.

Fig.S3. Autopsy of mice that were orally administered with water or the natural deep eutectic solvent (NADES). A&a, water treated group; B&b, the NADES treated group.

Fig.S4. Effect of the natural deep eutectic solvents (NADES) dilutions on the cell viability of MDCK-MDR1 cells (Mean ± SD, n = 3). MDCK-MDR1 cells were incubated with different NADES dilutions (0.1%, 0.3%, 1%, and 3%) for 4 or 24 h, respectively, and then cell viability was determined by the CCK-8 assay. **， p<0.01 vs control.

Fig.S5. Solubility of berberine hydrochloride (BBR) in water or solutions of malic acid (490.5 mg/mL), glucose (280.6 mg/mL), sucrose (517.7 mg/mL), choline chloride (19.0 mg/mL), respectively (Mean ± SD, n = 4). **， p<0.01 vs water.

Fig.S6. Solubility of berberine hydrochloride (BBR) in water or solutions (20, 40, 80, 160, 320 or 640 mg/mL) of malic acid, respectively (Mean ± SD, n = 4). **， p<0.01 vs water.

Fig.S7. Solubility of berberine hydrochloride (BBR, 20 mg/mL) in water (control, pH 3.57) or solutions of malic acid with different pH values, respectively (Mean ± SD, n = 4). **， p<0.01 vs Control.

Fig.S8. Survival curves of mice that were orally administered with malic acid solutions (n = 20). The mice were orally administered with 0.2 ml/10 g body weight the water solutions of malic acid. Then the death time of mice were recorded. Low, 49.0 mg/mL; Medium, 147.2 mg/mL; High, 490.0 mg/mL.

Fig.S9. Autopsy of mice that were orally administered with water or malic acid solutions. A&a, water treated group; B&b, malic acid (9.8 g/kg) treated group.

Fig.S10. Effect of water solutions of malic acid on the cell viability of MDCK-MDR1 cells (Mean ± SD, n = 3). MDCK-MDR1 cells were incubated with different water solution of malic acid (0.49, 1.47, or 4.90 mg/mL) for 4 or 24 h, respectively, and then cell viability was determined by the CCK-8 assay. **， p<0.01 vs control.

Fig.S11. ^{1}H NMR spectra of the natural deep eutectic solvent (NADES) and its D_2O dilutions (30%, 10%, 3%, 1%, 0.3%, and 0.1%).
